# Genetic evaluation with major genes and polygenic inheritance when some animals are not genotyped using gene content multiple-trait BLUP

**DOI:** 10.1186/s12711-015-0165-x

**Published:** 2015-11-17

**Authors:** Andrés Legarra, Zulma G. Vitezica

**Affiliations:** 1 INRA, UMR 1388 GenPhySE (Génétique, Physiologie et Systèmes d’Elevage), 31326 Castanet-Tolosan, France; 2 Université de Toulouse, INPT, ENSAT, UMR 1388 GenPhySE (Génétique, Physiologie et Systèmes d’Elevage), 31326 Castanet-Tolosan, France

## Abstract

**Background:**

In pedigreed populations with a major gene segregating for a quantitative trait, it is not clear how to use pedigree, genotype and phenotype information when some individuals are not genotyped. We propose to consider gene content at the major gene as a second trait correlated to the quantitative trait, in a gene content multiple-trait best linear unbiased prediction (GCMTBLUP) method.

**Results:**

The genetic covariance between the trait and gene content at the major gene is a function of the substitution effect of the gene. This genetic covariance can be written in a multiple-trait form that accommodates any pattern of missing values for either genotype or phenotype data. Effects of major gene alleles and the genetic covariance between genotype at the major gene and the phenotype can be estimated using standard EM-REML or Gibbs sampling. Prediction of breeding values with genotypes at the major gene can use multiple-trait BLUP software. Major genes with more than two alleles can be considered by including negative covariances between gene contents at each different allele. We simulated two scenarios: a selected and an unselected trait with heritabilities of 0.05 and 0.5, respectively. In both cases, the major gene explained half the genetic variation. Competing methods used imputed gene contents derived by the method of Gengler et al. or by iterative peeling. Imputed gene contents, in contrast to GCMTBLUP, do not consider information on the quantitative trait for genotype prediction. GCMTBLUP gave unbiased estimates of the gene effect, in contrast to the other methods, with less bias and better or equal accuracy of prediction. GCMTBLUP improved estimation of genotypes in non-genotyped individuals, in particular if these individuals had own phenotype records and the trait had a high heritability. Ignoring the major gene in genetic evaluation led to serious biases and decreased prediction accuracy.

**Conclusions:**

CGMTBLUP is the best linear predictor of additive genetic merit including pedigree, phenotype, and genotype information at major genes, since it considers missing genotypes. Simulations confirm that it is a simple, efficient and theoretically sound method for genetic evaluation of traits influenced by polygenic inheritance and one or several major genes.

## Background

Several major genes that influence quantitative traits in livestock species have been described, e.g. [1, 2]. Ignoring major genes in genetic evaluation affects estimation of breeding values [3]. Ideally, genes should be included as covariates in genetic evaluation models [4] but this is feasible only if all individuals are genotyped. If not all individuals are genotyped, there is no straightforward method to include observed genotypes at these genes in the genetic evaluation. The proposed methods are incomplete, impractical, and resort to approximations [5, 6], and sometimes focus more on estimation of gene effects rather than on genetic evaluation [6]. Algorithms of estimation that consider the joint distribution of genotypes at the major gene, polygenic components, and phenotypes include peeling and the Gibbs sampler. Exact peeling is unfeasible with animal pedigrees and approximations using iterative peeling [7] are inaccurate or even biased [8, 9], whereas use of the Gibbs sampler [10] is slow and its irreducibility is rarely guaranteed [11]. Alternatively, Gengler et al. [12, 13] suggested a practical method that considers gene content (the number of copies of a given allele carried by each individual) at a gene (or marker) as a quantitative trait and treats it within a best linear unbiased prediction (BLUP) framework. This approach allows estimation of gene content of ungenotyped individuals and its use as true genotype for genetic prediction. More recently, the idea of using gene content as a quantitative trait was taken up by Forneris et al. [14] for quality control of genotypes. However, if the major gene (e.g. *DGAT1*) has an effect on a quantitative trait recorded for genetic evaluation (e.g. fat content), it would make sense to analyze both “traits”, i.e. gene content at the major gene and the related quantitative trait, simultaneously as correlated traits. In this work, we show that the genetic correlation between a quantitative trait and gene content at a gene is a function of the effect of the gene on the trait. Furthermore, joint analysis within a BLUP framework results in an estimation method that is (1) computationally efficient, (2) theoretically sound (it is best, linear and unbiased in a classical sense) and therefore provides unbiased estimates of gene effects, and (3) uses information on the quantitative trait to infer the genotype at the gene for non-genotyped individuals. These features are absent in current procedures. Thus, we propose an integrated procedure for genetic evaluation of a complex trait (partially) controlled by a major gene and missing genotypes.

## Methods

### Theory

#### Gene content as a quantitative trait

Gene content ($z$) at a gene is the number of copies of a particular reference allele (e.g. *z* = 0, 1 or 2 for AA, AB and BB, respectively) [15]. It can be considered as a quantitative trait, with the mean in the base population equal to 2*p* (*p* is the allele frequency in the base population) and variance equal to 2*pq* where $q = 1 - p$. The covariance between gene contents at the major gene for two individuals is $Cov(z_{i} ,z_{j} ) = A_{ij} 2pq$ [[16], Equation (8)], where $A_{ij}$ is the additive relationship between individuals *i* and *j*. This leads naturally to the construction of linear estimators of genotypes [13, 17], estimation of base population allele frequencies [18] and quality control checks [14]. For all these cases, a linear model of the form ${\mathbf{z}} = {\mathbf{X}}_{z} {\mathbf{b}}_{z} + {\mathbf{W}}_{z} {\mathbf{u}}_{z} + {\mathbf{e}}_{z}$ is used, where ${\mathbf{X}}_{z}$ is typically a column of 1s for a general mean, ${\mathbf{b}}_{z} = 2p$ (although multiple base populations can be considered as well), ${\mathbf{u}}_{z}$ includes genetic values for gene content ($Cov({\mathbf{u}}_{z} ) = {\mathbf{A}}2pq$) expressed as the deviation of each individual from the base population mean, ${\mathbf{W}}_{z}$ is an incidence matrix for genotyped individuals, and ${\mathbf{e}}_{z}$ is a vector of error terms that should be equal to 0 but, in practice, $\sigma_{{e_{z} }}^{2}$ in $Var({\mathbf{e}}_{z} ) = {\mathbf{I}}\sigma_{{e_{z} }}^{2}$ is assigned a very small value, which allows some genotyping errors and the use of mixed model equations for estimation.

#### Covariance between gene content at the major gene and the quantitative trait

Imagine that the gene coded in ${\mathbf{z}}$ as $\left\{ {0,\;1,\;2} \right\}$ has an effect on the trait, such as $\left\{ {0,\;\alpha ,\;2\alpha } \right\}$ [*α* can be understood as a substitution effect, possibly including non-additive gene action; for instance, if dominance exists, the substitution effect is $\alpha = a + d(q - p)$ where $a$ is half the distance between the two genotypic values of the homozygous individuals and $d$ the genotypic value of the heterozygote [15]]. We will assume the substitution effect to be constant across generations, which holds if there is no interaction with the environment, if the gene is the true causal variant, and if allelic frequencies do not change. Consider a vector of phenotypes for the quantitative trait *y* determined partly by additive genetic effects ${\mathbf{u}}_{y}$. The additive genetic effects can be decomposed as:

$${\mathbf{u}}_{y} =\varvec{\varepsilon}+ {\mathbf{u}}_{y}^{*} =\varvec{\varepsilon}+ {\mathbf{z}}\alpha - E({\mathbf{z}})\alpha =\varvec{\varepsilon}+ {\mathbf{u}}_{z} \alpha ,$$

i.e. decomposed in a polygenic component $\varvec{\varepsilon}$ and a component due to the effect of the major gene, ${\mathbf{u}}_{y}^{*} = {\mathbf{u}}_{z} \alpha$ (because the heritability of gene content is 1, ${\mathbf{u}}_{z}$ is simply ${\mathbf{z}}$ minus its average). For instance, for four individuals that have genotypes $\left\{ {AA,\;AB,\;AA,\;BB} \right\}$ with a base population allele frequency of 0.5, the decomposition of the total additive genetic value would be:

$$\left( {\begin{array}{*{20}c} {u_{y1} } \\ {u_{y2} } \\ {u_{y3} } \\ {u_{y4} } \\ \end{array} } \right) = \left( {\begin{array}{*{20}c} {\varepsilon_{1} } \\ {\varepsilon_{2} } \\ {\varepsilon_{3} } \\ {\varepsilon_{4} } \\ \end{array} } \right) + \left( {\begin{array}{*{20}c} {u_{y1}^{*} } \\ {u_{y2}^{*} } \\ {u_{y3}^{*} } \\ {u_{y4}^{*} } \\ \end{array} } \right) = \left( {\begin{array}{*{20}c} {\varepsilon_{1} } \\ {\varepsilon_{2} } \\ {\varepsilon_{3} } \\ {\varepsilon_{4} } \\ \end{array} } \right) + \left( {\begin{array}{*{20}c} { - 1} \\ 0 \\ { - 1} \\ 1 \\ \end{array} } \right)\alpha .$$

The polygenic component $\varvec{\varepsilon}$ is assumed to follow a multivariate normal distribution with mean zero and $Var(\varvec{\varepsilon}) = {\mathbf{A}}\sigma_{\varepsilon }^{2}$.

The genetic variance of gene content is $\sigma_{{u_{z} }}^{2} = 2pq$. The genetic variance of $u_{y}^{*}$ (the genetic variance of the quantitative trait $y$ explained by the gene) is $\sigma_{{u_{y}^{*} }}^{2} = 2pq\alpha^{2}$ as expected, and the total additive genetic variance is $\sigma_{{u_{y} }}^{2} = \sigma_{\varepsilon }^{2} + 2pq\alpha^{2}$. Accordingly, $Var({\mathbf{u}}_{y}^{*} ) = {\mathbf{A}}2pq\alpha^{2}$. Thus, $Var({\mathbf{u}}_{y} ) = Var(\varvec{\varepsilon}+ {\mathbf{u}}_{\varvec{y}}^{\varvec{*}} ) = {\mathbf{A}}\sigma_{\varepsilon }^{2} + {\mathbf{A}}2pq\alpha^{2} = {\mathbf{A}}(\sigma_{\varepsilon }^{2} + 2pq\alpha^{2} ) = {\mathbf{A}}\sigma_{{u_{y} }}^{2}$. We assume that, in the base population, there is no correlation between the gene content and the polygenic background [$Cov\left( {\varepsilon,{z}} \right) = 0$]. For the same reason, the genetic covariance between the two traits (quantitative trait *y* and gene content *z*) in the base population is $\sigma_{{u_{z,y} }} = Cov({{u}}_{y} ,{{u}}_{z} ) = Cov({{u}}_{y}^{*} ,{{u}}_{z} ) = Cov({{u}}_{z} \alpha ,{{u}}_{z} ) = Var({{u}}_{z} )\alpha = 2pq\alpha$. Thus, the genetic covariance between gene content at a gene and a quantitative trait is simply the heterozygosity at the gene times the substitution effect of the gene on the trait.

We can now write the matrix of covariances between breeding values for gene content, ${\mathbf{u}}_{z}$, and total breeding value for trait $y$, ${\mathbf{u}}_{y}$. This matrix of covariances is the second moment of their joint distribution, which holds even if the distribution of ${\mathbf{z}}$ (and ${\mathbf{u}}_{z}$) is non-normal.

$$Cov\left( {\begin{array}{*{20}c} {{\mathbf{u}}_{y} } \\ {{\mathbf{u}}_{z} } \\ \end{array} } \right) = \left( {\begin{array}{*{20}c} {{\mathbf{A}}\sigma_{{u_{y} }}^{2} } & {{\mathbf{A}}\sigma_{{u_{z,y} }} } \\ {{\mathbf{A}}\sigma_{{u_{z,y} }} } & {{\mathbf{A}}\sigma_{{u_{z} }}^{2} } \\ \end{array} } \right) = \left( {\begin{array}{*{20}c} {{\mathbf{A}}(\sigma_{\varepsilon }^{2} + 2pq\alpha^{2} )} & {{\mathbf{A}}2pq\alpha } \\ {{\mathbf{A}}2pq\alpha } & {{\mathbf{A}}2pq} \\ \end{array} } \right) = {\mathbf{G}}_{0} \otimes {\mathbf{A}},$$

with

$${\mathbf{G}}_{0} = \left( {\begin{array}{*{20}c} {\sigma_{{u_{y} }}^{2} } & {\sigma_{{u_{z,y} }} } \\ {\sigma_{{u_{z,y} }} } & {\sigma_{{u_{z} }}^{2} } \\ \end{array} } \right) = \left( {\begin{array}{*{20}c} {(\sigma_{\varepsilon }^{2} + 2pq\alpha^{2} )} & {2pq\alpha } \\ {2pq\alpha } & {2pq} \\ \end{array} } \right)$$

with inverse

$${\mathbf{G}}_{0}^{ - 1} = \left( {\begin{array}{*{20}c} {\sigma^{11} } & {\sigma^{12} } \\ {\sigma^{21} } & {\sigma^{22} } \\ \end{array} } \right) = \left( {\begin{array}{*{20}c} {1/\sigma_{\varepsilon }^{2} } & { - \alpha /\sigma_{\varepsilon }^{2} } \\ { - \alpha /\sigma_{\varepsilon }^{2} } & {\frac{{\sigma_{\varepsilon }^{2} + 2pq\alpha^{2} }}{{2pq\sigma_{\varepsilon }^{2} }}} \\ \end{array} } \right).$$

This enables a multiple-trait evaluation using the observed trait phenotypes and the observed genotypes to be performed. Assuming a small residual variance for gene content, it is possible to use Henderson’s mixed model equations, which are for a simple case (no fixed effects, one record per individual) equal to:

$$\left( {\begin{array}{*{20}l} {{\mathbf{I}}\sigma_{ey}^{ - 2} + {\mathbf{A}}^{ - 1} \sigma^{11} } \hfill & {{\mathbf{A}}^{ - 1} \sigma^{12} } \hfill \\ {{\mathbf{A}}^{ - 1} \sigma^{21} } \hfill & {{\mathbf{A}}^{ - 1} \sigma^{22} + \, \varvec{I}\sigma_{ez}^{ - 2} } \hfill \\ \end{array} } \right)\left( {\begin{array}{*{20}l} {\widehat{{\mathbf{u}}}_{y} } \hfill \\ {\widehat{{\mathbf{u}}}_{z} } \hfill \\ \end{array} } \right) = \left( {\begin{array}{*{20}l} {{\mathbf{y}}\sigma_{ey}^{ - 2} } \hfill \\ {{\mathbf{z}}\sigma_{ez}^{ - 2} } \hfill \\ \end{array} } \right)$$

where $\sigma_{ey}^{2}$ is the residual variance of the observed trait phenotype and $\sigma_{ez}^{2}$ the residual variance of the observed genotypes (typically a value very close to 0). This two-trait model of ${\mathbf{y}}$ and ${\mathbf{z}}$ as phenotypes is another way of writing the “mixed models” that are used in association studies (EMMAX and related models [19, 20], which go back to [4]), in which the effect of the gene is fitted as a covariate. This model is:

$${\mathbf{y}} = \cdots + {\mathbf{z}}\alpha +\varvec{\varepsilon}+ {\mathbf{e}}$$

where $\cdots$ implies other effects (e.g., sex, herd-year-season or permanent environment) and ${\mathbf{z}}$ contains the genotypes for the locus considered. Our particular case considers that pedigrees are available and $Var(\varvec{\varepsilon}) = {\mathbf{A}}\sigma_{\varepsilon }^{2}$, using a kinship matrix from pedigree, as in [4]. Note that if $z$ is observed, and because its heritability is equal to 1, it is irrelevant to consider that a priori it has a covariance matrix ${\mathbf{A}}2pq$. A proof that our multiple-trait model as described above and the model in [4] are equivalent models is given in the “Appendix”.

#### Estimation of effects of a single gene when some genotypes are missing

However, implementing the model in [4] in which the effect of the gene is fitted as a covariate requires that all genotypes in ${\mathbf{z}}$ are observed. To remove this requirement, Gengler et al. [13] suggested using estimates of ${\mathbf{z}}$ in place of true values, although Christensen and Lund [17] remarked that this ignores uncertainty in the estimation of ${\mathbf{z}}$ and showed how to include this uncertainty in a single-step procedure. In this paper, we suggest an extension of both these ideas.

The real interest of our proposed method is in the case of missing genotypes and/or phenotypes (e.g. bulls are genotyped and dairy cows have phenotypes for milk yield), a situation (several traits with missing records) that is frequent in animal breeding [21, 22] but has only been recently considered in human genetics [23]. In the case of missing records for either $y$, $z$, or both, the covariance matrix of ${\mathbf{y}}$ and ${\mathbf{z}}$ can be rewritten as [22]:

$$Cov\left( {\begin{array}{*{20}c} {\mathbf{y}} \\ {\mathbf{z}} \\ \end{array} } \right) = {\mathbf{W}}\left( {{\mathbf{G}}_{0} \otimes {\mathbf{A}}} \right){\mathbf{W}}^{'} + {\mathbf{R}}$$

where $${\mathbf{W}} = \left( {\begin{array}{*{20}c} {{\mathbf{W}}_{y} } & 0 \\ 0 & {{\mathbf{W}}_{z} } \\ \end{array} } \right)$$ is an incidence matrix relating individuals to ${\mathbf{y}}$ and ${\mathbf{z}}$ (and which specifically keeps track of the missing observations) and **R** is a block-diagonal matrix that contains residual variances for ${\mathbf{y}}$, for ${\mathbf{z}}$, or for both, depending on the pattern of missingness of records. Note that if all ${\mathbf{z}}$ are observed, and because it has a heritability of 1, there is no uncertainty associated with ${\mathbf{z}}$ and the model is equivalent to the regression of Kennedy et al. [4]. Assuming normality for ${\mathbf{z}}$, the covariance presented above also defines a likelihood function, i.e.:

$$p\left( {\begin{array}{*{20}c} {\mathbf{y}} \\ {\mathbf{z}} \\ \end{array} \Big|{\mathbf{G}}_{0} ,{\mathbf{R}},{\mathbf{b}}_{y} ,{\mathbf{b}}_{z} } \right) \approx N\left( {\begin{array}{*{20}c} {{\mathbf{X}}_{y} {\mathbf{b}}_{y} } \\ {{\mathbf{X}}_{z} {\mathbf{b}}_{z} } \\ \end{array} ,{\mathbf{W}}\left( {{\mathbf{G}}_{0} \otimes {\mathbf{A}}} \right){\mathbf{W}}^{'} + {\mathbf{R}}} \right).$$

This covariance structure is correct if missingness is ignorable in the sense of Rubin [24], that is, the pattern of missingness does not depend on specific values of ${\mathbf{y}}$ and ${\mathbf{z}}$. For instance, when only sick animals are genotyped, the pattern of missingness is not ignorable and thus the above likelihood is not correct. Also, if only elite animals are genotyped, there will be a positive residual covariance between phenotype for the trait and gene content.

If missingness can be ignored, any pattern of ignorable missingness of $y$ and $z$ can be considered and, thus, all individuals can be included in the analysis, regardless of whether they have observations only for the phenotype, only for the genotype, both, or none. The normal multivariate likelihood is an approximation as we assume normality for ${\mathbf{z}}$. Using this framework, two different analyses can be performed.

#### Gene content multiple-trait BLUP (GCMTBLUP)

The variance components in ${\mathbf{G}}_{0}$ can be constructed from estimates of the gene effect and its allele frequencies at the base population. Alternatively, they can be estimated from the analysis that we present in the next section (GCMTREML).

Assuming the variance components in ${\mathbf{G}}_{0}$ are known, a multiple-trait analysis can be run, where the first trait is $y$ and the second trait is $z$. The model for $y$ does not explicitly include the gene, which is included through genetic covariances of $y$ with $z$. The model for $z$ includes an overall mean (or several if there are several genetic origins) plus the additive breeding values ${\mathbf{u}}_{z}$. For instance:

$${\mathbf{y}} = {\mathbf{X}}_{y} {\mathbf{b}}_{y} + {\mathbf{W}}_{y} {\mathbf{u}}_{y} + {\mathbf{e}}_{y}$$

$${\mathbf{z}} = {\mathbf{X}}_{z} {\mathbf{b}}_{z} + {\mathbf{W}}_{z} {\mathbf{u}}_{z} + {\mathbf{e}}_{z}$$

with genetic covariance between traits as described above:

$${\mathbf{G}}_{0} = \left( {\begin{array}{*{20}c} {\sigma_{{u_{y} }}^{2} } & {\sigma_{{u_{z,y} }} } \\ {\sigma_{{u_{z,y} }} } & {\sigma_{{u_{z} }}^{2} } \\ \end{array} } \right) = \left( {\begin{array}{*{20}c} {\left( {\sigma_{\varepsilon }^{2} + 2pq\alpha^{2} } \right)} & {2pq\alpha } \\ {2pq\alpha } & {2pq} \\ \end{array} } \right).$$

In addition, ${\mathbf{e}}_{y}$ and ${\mathbf{e}}_{z}$ are uncorrelated, and $\sigma_{{e_{z} }}^{2}$ must be set to a small but non zero value in order to use the mixed model equations. Based on these equations, GCMTBLUP can deal with any pattern of missing traits and be easily solved with existing software. Solutions (BLUP) of ${\mathbf{u}}_{y}$ and ${\mathbf{u}}_{z}$ contain estimates of the overall breeding value for trait $y$, which include the effect of the major gene, and estimates of the breeding value for $z$, that is, estimates of gene content. Estimates of the polygenic component of ${\mathbf{u}}_{y}$ can be obtained as $\widehat{\boldsymbol{\varepsilon} } = \widehat{{{\mathbf{u}}_{y} }} - \widehat{{{\mathbf{u}}_{z} }}\alpha$. Note that these solutions are obtained regardless of the missing data pattern; thus, an individual with no genotype data but with phenotype data will benefit from own phenotype records and from the genotype and phenotype records of all its relatives. Accordingly, an individual with no phenotype record but with genotype data will be predicted based on the phenotypes of the relatives and its own genotype data. To date, there is no efficient method that can combine both sources of information with missing data. Because the heritability of $z$ is equal to 1, the genomic estimated breeding value of genotyped individuals is equal to $z$ minus its base population average. The method can in principle be applied to as many genes and traits as needed, although complexity of the system of equations will grow up quadratically.

#### Gene content multiple-trait REML (GCMTREML)

A simple way to estimate the substitution gene effect ($\alpha$), its heterozygosity ($2pq$) and the polygenic variance not accounted for by the trait ($\sigma_{\varepsilon }^{2}$) is to estimate the genetic covariance matrix ${\mathbf{G}}_{0}$. This estimation can typically be carried by multiple-trait restricted maximum likelihood (REML) [22, 25, 26]. REML is a consistent estimator under normality, but also has good properties as a minimum-variance quadratic unbiased estimator (MIVQUE) under non normality [27]. Alternatively, a Bayesian estimator using a multiple-trait Gibbs sampler can be used.From the estimated covariance matrix:

$$\widehat{{\mathbf{G}}}_{0} = \left( {\begin{array}{*{20}c} {\widehat{\sigma }_{{u_{y} }}^{2} } & {\widehat{\sigma }_{{u_{z,y} }} } \\ {\widehat{\sigma }_{{u_{z,y} }} } & {\widehat{\sigma }_{{u_{z} }}^{2} } \\ \end{array} } \right) = \left( {\begin{array}{*{20}c} {(\widehat{\sigma_{\varepsilon }^{2}} + \widehat{{2pq\alpha^{2} }})} & {\widehat{2pq\alpha }} \\ {\widehat{2pq\alpha }} & {\widehat{2pq}} \\ \end{array} } \right)$$

the estimate of the gene effect is $\widehat{\alpha } = \widehat{\sigma }_{{u_{z,y} }} /\widehat{\sigma }_{{u_{z} }}^{2}$. A confidence interval for this estimate can be obtained by the delta method [28]. Because it is derived from the joint covariances of the trait and gene content, this estimate $\widehat{\alpha }$ should provide unbiased estimates of the gene effect.

Significance of the gene effect can be tested by a likelihood ratio test between this alternative model, for which $\sigma_{{u_{z,y} }}$ is estimated, and a null model in which $\sigma_{{u_{z,y} }} = 0$ but in which all other variances are also estimated by REML. This test is asymptotically distributed as a Chi square with one degree of freedom since $\sigma_{{u_{z,y} }}$ is unbounded [28], contrary to the typical case in genetics where the variance component is bounded [29, 30].

This analysis also results in an estimate of the allele frequency of the major gene in the base generation, from $\widehat{\sigma }_{{u_{z} }}^{2} = 2\widehat{p}(1 - \widehat{p})$. The allele frequency can also be estimated from the overall mean of the model, $\widehat{b}_{z} = 2\widehat{p}$. In practice, they are close but not exactly the same.

#### GCMTBLUP with a multiallelic gene

Consider a locus with multiple *n* alleles $(1, 2 \ldots n)$, with respective effects $\alpha_{1} , \ldots ,\alpha_{n}$. In this case, the gene content of each allele can be considered as a “trait”, with value 0, 1 or 2 if the allele is not observed, observed once, or observed twice, respectively [16]. Thus, individuals with genotypes 11, 12, 33 would have gene contents $z_{1}$, $z_{2}$, $z_{3}$ with values $$\left( {\begin{array}{*{20}c} 2 \\ 1 \\ 0 \\ \end{array} } \right),\left( {\begin{array}{*{20}c} 0 \\ 1 \\ 0 \\ \end{array} } \right),{\text{and}}\left( {\begin{array}{*{20}c} 0 \\ 0 \\ 2 \\ \end{array} } \right)$$, respectively.

Obviously, these three pseudo-traits are correlated, because absence of alleles 1 and 2 implies that the genotype is 33. This corresponds to two times a multinomial distribution with parameters $\left( {p_{1} ,p_{2} , \ldots p_{n} } \right)$, the allele frequencies, and the covariance matrix between gene contents is singular and equal to:

$$Var\left( {\begin{array}{*{20}c} {z_{1} } \\ {z_{2} } \\ \cdots \\ {z_{n} } \\ \end{array} } \right) = 2\left( {\begin{array}{*{20}c} {p_{1} q_{1} } & { - p_{1} p_{2} } & \cdots & { - p_{1} p_{n} } \\ { - p_{2} p_{1} } & {p_{2} q_{2} } & \cdots & { - p_{2} p_{n} } \\ \cdots & \cdots & \cdots & \cdots \\ { - p_{n} p_{1} } & { - p_{n} p_{2} } & \cdots & {p_{n} q_{n} } \\ \end{array} } \right).$$

Thus, inclusion of gene contents in the model to account for the different effects of each allele can be done by using a multiple-trait model that is similar to the model already described for a biallelic gene but including the quantitative trait $y$ and $n - 1$ gene contents $z$ (one of the alleles at the gene is ignored to avoid redundancy). Therefore, a genetic covariance matrix of size *n* is estimated (of which the above matrix is a submatrix). The covariance matrix of $n - 1$ dimension generalizes the idea of Gengler et al. [13] for biallelic loci to multiallelic loci. Gengler et al. [13] and Mulder et al. [31] suggested fitting each allele separately but this ignores that presence of one allele impedes presence of another one. The use of genetic covariances among gene contents is more accurate. The effect of the ignored allele is absorbed into the overall mean of the model (and thus implicitly set to 0). The effect $\alpha_{i}$ of the i-th allele is then estimated as $\widehat{\alpha }_{i} = \widehat{\sigma }_{{u_{{z_{i} ,y}} }} /\widehat{\sigma}_{{u_{{z_{i} }} }}^{2}$.

#### Major genes on sex chromosomes

Some major genes lie on the X chromosome, with males having one copy and females two copies; one example is the gene *BMP15* [1]. In this case, the covariance of gene contents between two individuals does not follow regular relationships. However, the relationship matrix of X-chromosomal inheritance [32] correctly describes the covariances of gene contents on the X chromosome between two individuals and also the covariance between the quantitative trait $y$ and the observed genotype $z$, and this relationship matrix can be easily used in the GCMTBLUP analysis.

#### Numerical example

Assume pedigree and phenotype as in Table 1. The two vectors of traits are ${\mathbf{y}} = \left( {NA,\;15,\;7,\;NA,\;12} \right)^{{\prime }}$ (where NA codes for “missing”) and ${\mathbf{z}} = (1,\;NA,\;NA,\;2,\;0)^{{\prime }}$, where $z$ is the number of B alleles. The respective incidence matrices are (for the general means):

$${\mathbf{X}}_{\text{y}} = \left( {\begin{array}{*{20}c} 0 & 1 & 1 & 0 & 1 \\ \end{array} } \right)^{{\prime }} \quad{\text{and}}\quad {\mathbf{X}}_{\text{z}} = \left( {\begin{array}{*{20}c} 1 & 0 & 0 & 1 & 1 \\ \end{array} } \right)^{{\prime }} ,$$

and (for the breeding values):

$${\mathbf{W}}_{\text{y}} = \left( {\begin{array}{*{20}c} 0 & 0 & 0 & 0 & 0 \\ 0 & 1 & 0 & 0 & 0 \\ 0 & 0 & 1 & 0 & 0 \\ 0 & 0 & 0 & 0 & 0 \\ 0 & 0 & 0 & 0 & 1 \\ \end{array} } \right),$$

$${\text{and}}\quad{\mathbf{W}}_{z} = \left( {\begin{array}{*{20}c} 1 & 0 & 0 & 0 & 0 \\ 0 & 0 & 0 & 0 & 0 \\ 0 & 0 & 0 & 0 & 0 \\ 0 & 0 & 0 & 1 & 0 \\ 0 & 0 & 0 & 0 & 1 \\ \end{array} } \right).$$

Assume the residual covariances $${\mathbf{R}}_{0} = \left( {\begin{array}{*{20}c} {0.95} & 0 \\ 0 & {0.001} \\ \end{array} } \right)$$ and $${\mathbf{G}}_{0} = \left( {\begin{array}{*{20}c} {0.05} & {0.11} \\ {0.11} & {0.5} \\ \end{array} } \right),$$ implying a quantitative trait with a heritability of 0.05 and half of the genetic variation explained by a single gene with an allele frequency of 0.5. Note that heritability for gene content is equal to $0.05/(0.001 + 0.5) \approx 0.998$, which is not exactly 1, to allow the use of Henderson’s mixed model equations.

**Table 1:** Example pedigree and data

Individual	Father	Mother	Phenotype	Genotype
1	0	0	Missing	AB
2	0	0	15	Missing
3	1	2	7	Missing
4	1	2	Missing	BB
5	1	2	12	AA

With these elements, the mixed model equations can be created. Assume that in the mixed model equations, traits are ordered within levels of effects, that is, mixed model equations contain ${\mathbf{A}}^{ - 1} \otimes {\mathbf{G}}_{0}^{ - 1}$ instead of ${\mathbf{G}}_{0}^{ - 1} \otimes {\mathbf{A}}^{ - 1}$. The right hand side is:

$$\left( {35.79, \;3000,\; 0,\; 1000, \;15.79,\; 0,\; 7.37,\; 0, \;0, \;2000, \;12.63, \,0} \right)^{{\prime }} .$$

For example, the two first values are scaled sums of phenotypes and genotypes. The following two values contain the phenotype and the genotype of animal 1; the next two for animal 2; and so on.

The left hand side is (truncated at two decimal places):

$$\begin{array}{*{20}c} {3.15} & 0 & 0 & 0 & {1.05} & 0 & {1.05} & 0 & 0 & 0 & {1.05} & 0 \\ 0 & {3000} & 0 & {1000} & 0 & 0 & 0 & 0 & 0 & {1000} & 0 & {1000} \\ 0 & 0 & {96.89} & { - 21.31} & {58.13} & { - 12.79} & { - 38.75} & {8.52} & { - 38.75} & {8.52} & { - 38.75} & {8.52} \\ 0 & {1000} & { - 21.31} & {1009.68} & { - 12.79} & {5.81} & {8.52} & { - 3.87} & {8.52} & { - 3.87} & {8.52} & { - 3.87} \\ {1.05} & 0 & {58.13} & { - 12.79} & {97.95} & { - 21.31} & { - 38.75} & {8.52} & { - 38.75} & {8.52} & { - 38.75} & {8.52} \\ 0 & 0 & { - 12.79} & {5.81} & { - 21.31} & {9.68} & {8.52} & { - 3.87} & {8.52} & { - 3.87} & {8.52} & { - 3.87} \\ {1.05} & 0 & { - 38.75} & {8.52} & { - 38.75} & {8.52} & {78.57} & { - 17.05} & 0 & 0 & 0 & 0 \\ 0 & 0 & {8.52} & { - 3.87} & {8.52} & { - 3.87} & { - 17.05} & {7.75} & 0 & 0 & 0 & 0 \\ 0 & 0 & { - 38.75} & {8.52} & { - 38.75} & {8.52} & 0 & 0 & {77.51} & { - 17.05} & 0 & 0 \\ 0 & {1000} & {8.52} & { - 3.87} & {8.52} & { - 3.87} & 0 & 0 & { - 17.05} & {1007.75} & 0 & 0 \\ {1.05} & 0 & { - 38.75} & {8.52} & { - 38.75} & {8.52} & 0 & 0 & 0 & 0 & {78.57} & { - 17.05} \\ 0 & {1000} & {8.52} & { - 3.87} & {8.52} & { - 3.87} & 0 & 0 & 0 & 0 & { - 17.03} & {1007.75} \\ \end{array}$$

Solutions to the equations are ($$\begin{array}{*{20}c} { 1 1. 4 2} & { 1.0 5} & { - 0.0 6} & { - 0.0 5} & {0.0 6} & {0.0 5} & { - 0. 1 1} & { - 0. 2 5} & {0. 2 1} & {0. 9 4} & { - 0. 2 2} & { - 1.0 5} \\ \end{array}$$). Note that 1.05 is the estimate of $2p$ and estimated breeding values for $z$ for individuals 1, 4 and 5 are equal to −0.05, 0.94 and −1.05, respectively, which summed to the mean of the trait give the observed genotypes 1, 2 and 0.

### Simulations

#### Scenario with a major gene for a lowly heritable trait

Consider the following scenario with a major gene for a lowly heritable trait. This is loosely based on the example of prolificacy genes for sheep [1]. The rationale for this scenario is that a major gene is typically fixed by selection, unless selection is recent, not strong, or the trait has a low heritability. Therefore, to simulate a major gene that has undergone selection but not fixation (yet), we used QMSim [33] under a scenario similar to selection for litter size in sheep: heritability of 0.05, of which half (0.025) is due to the major gene and the rest is polygenic. Selection is based on progeny-testing for males (with 10 to 20 daughters per male). Eleven generations with 2600 animals each were simulated, with 200 males acting as sires. The gene allele frequency at generation 0 was set to 0.5, resulting in an effect of $\alpha = \sqrt {0.025/(2 \cdot 0.5^{2} )} = 0.226$ in the base population for a phenotypic variance of 1. Phenotypes (for a continuous trait) were recorded for the first 10 generations, with animals in generation 11 used as candidates for selection. Only females were phenotyped and all 920 sires were genotyped. In addition, two scenarios were simulated for 1315 selection candidates from generation 11; they were considered as genotyped in one scenario but not in the other.

Genetic parameters of gene content and the phenotypic trait were estimated based on the GCMTREML method described previously using the EM-REML algorithm of remlf90 [34], and based on these variance component estimates, the effect of the gene was derived. Using these estimates, breeding values for the continuous trait and for gene content were obtained for all individuals using GCMTBLUP. The same process was repeated by setting to 0 the genetic covariance between the continuous trait and the gene content, which prevents observed genotypes from influencing estimation of breeding values and trait phenotypes from influencing the prediction of gene content. The simulation was repeated 10 times. A particular case that was also studied, which represents the upper bound of accuracy, is when all individuals are genotyped. Quality of prediction was assessed for the 1315 candidates to selection (generation 11) and for the 2800 females of generation 10, that had own phenotypes but no genotype data. We checked accuracy and bias [slope of the regression of true breeding values (TBV) on estimated breeding values (EBV)] for the quantitative trait and for the prediction of gene content, i.e., of the genotype of the candidate (which may be useful, e.g., for planned matings).

In addition to joint analysis of all genotypes and phenotypes, we used two methods that first estimated genotypes and then used the estimated genotypes as true genotypes fitted explicitly as a covariable [4]. We implemented the method of Gengler et al. [13], in which gene content was predicted as a quantitative trait for ungenotyped animals based on observed genotypes for genotyped animals. The second method was “peeling” [35], which considers the joint distribution of all observed genotypes conditional on the pedigree and takes the discrete nature of alleles into account (i.e., homozygous parents can only transmit one allele). We used iterative peeling as described in Fernando et al. [35], which results in posterior probabilities of each genotype for each individual. These were combined to create a prediction of gene content. In contrast to the prediction of gene content of Gengler et al. [13], iterative peeling is nonlinear and, in principle, is more accurate, although in the presence of pedigree loops it does not produce the optimal solutions that are obtained by exact peeling [35].

#### Scenario with a major gene for a highly heritable trait

The second scenario was as above, but the trait was highly heritable i.e. 0.5, of which half was explained by the major gene. The trait was unselected to avoid fixation of the favorable allele of the major gene. The aim was to reflect major genes such as *DGAT1* for which both alleles are selected or, alternatively, a major gene for a trait that has not been selected so far.

## Results

Estimation of the variance component associated to the major gene effect took a large number of iterations (around 100, totaling 30 min until convergence) in the EM algorithm used for REML, since the likelihood was rather flat (animals with genotypes did not have phenotypes and vice versa). Using average information REML (AIREML; [26]) did not improve convergence since heritability of gene content is in the boundary of the parametric space. Although estimation of the genetic parameters was slow, GCMTBLUP prediction with known variance components was very fast; a simulation similar to the low heritability scenario that included 10 million females non genotyped but with records and 50,000 genotyped males without records was solved in 45 min, with 300 iterations using preconditioned conjugate gradients and iteration on data (blup90iod2: [34]). Single-trait estimation without genotypes took 25 min and 155 iterations.

### Scenario with a major gene for a lowly heritable trait

Using GCMTREML, estimates of the substitution effect were equal to 0.237 ± 0.03 and 0.223 ± 0.03, when generation 11 was genotyped or not, respectively. These results compare very well with the true value of 0.226. In all cases, the likelihood ratio test supported the hypothesis of non-null genetic covariance, that is, of a non-null effect of the major gene. Heritability was slightly underestimated (0.04 instead of 0.05), possibly due to selection.

Using the method of Gengler et al. [13] to estimate genotypes and then fitting them as known, resulted in estimates of gene effect of 0.269 ± 0.03 when animals in generation 11 were genotyped, and 0.250 ± 0.03 when they were not. Using iterative peeling, the estimates were equal to 0.281 ± 0.03 and 0.260 ± 0.03, respectively. Thus, these two methods resulted in estimated gene effects that were biased upward. Selection for the favorable allele is unaccounted for in these methods and this may be a cause of bias. Another possible source of bias is the use of estimated genotypes (estimated without including phenotype information).

Prediction accuracies of selection candidates are in Table 2 and in all cases, GCMTBLUP, the method of Gengler et al. [13] and iterative peeling were more accurate and less biased than regular BLUP, even when selection candidates were not genotyped. In general, differences between methods were significant (p < 0.05) for the slope but not for accuracy but GCMTBLUP was generally the best. GCMTBLUP and the other methods are more accurate than simple BLUP because (1) the overall genetic value of their ancestors is better modeled, and (2) the genotype at the major genes in the candidates is considered. These two advantages are cumulative, based on comparing accuracy when candidates are genotyped versus not genotyped. When candidates were genotyped, the method of Gengler et al. [13] and peeling were as accurate as GCMTBLUP but were more biased and resulted in EBV that showed over-dispersion. All studied methods were clearly superior to simple BLUP. Regarding prediction of genotypes, all methods had similar accuracies and slopes. If the gene effect was accurately estimated with partial genotyping, as for GCMTBLUP, selection candidates did not benefit, in terms of accuracy, from extending the genotyping to all the population.

**Table 2:** Low heritability scenario for candidates to selection

Method	Quantitative trait		Gene content		Candidates genotyped
	Accuracy	Slope	Accuracy	Slope	
Simple BLUP	0.30 ± 0.04	0.86 ± 0.13	–	–	
GCMTBLUP	0.39 ± 0.02	0.98 ± 0.13	0.53 ± 0.05	1.00 ± 0.05	No
Method of Gengler et al.	0.39 ± 0.02	0.94 ± 0.11	0.52 ± 0.05	1.00 ± 0.05	No
Iterative peeling	0.39 ± 0.02	0.92 ± 0.04	0.53 ± 0.05	0.97 ± 0.06	No
GCMTBLUP	0.59 ± 0.03	0.95 ± 0.05	1	1	Yes
Method of Gengler et al.	0.59 ± 0.04	0.87 ± 0.05	1	1	Yes
Iterative peeling	0.59 ± 0.04	0.83 ± 0.04	1	1	Yes
All genotyped^a^	0.59 ± 0.03	0.98 ± 0.06	1	1	

Accuracy, intercept and slope of the comparison of estimated and true breeding values of 1315 selection candidates to selection for the model including gene content (GCMTBLUP) or not (Simple BLUP), for the method of Gengler et al. [13], and for the iterative peeling method of gene prediction

^a^All individuals are genotyped

Results for females with own phenotype were similar (Table 3). Again, GCMTBLUP was slightly more accurate and less biased than the other methods that used genotypes. Females benefitted from the genotyping of selection candidates because there was slightly more information. However, females benefitted from own genotyping data, even if they had own phenotype records, because the trait had a low heritability and thus did not provide much information on the genotype, although the gene explained half the genetic variance.

**Table 3:** Low heritability scenario for females with phenotype

Method	Quantitative trait		Gene content		Candidates genotyped
	Accuracy	Slope	Accuracy	Slope	
Simple BLUP	0.36 ± 0.04	0.85 ± 0.09	–	–	
GCMTBLUP	0.43 ± 0.04	0.94 ± 0.09	0.53 ± 0.05	0.98 ± 0.05	No
Method of Gengler et al.	0.42 ± 0.04	0.94 ± 0.08	0.52 ± 0.05	0.98 ± 0.06	No
Iterative peeling	0.42 ± 0.04	0.92 ± 0.07	0.52 ± 0.05	0.96 ± 0.06	No
GCMTBLUP	0.45 ± 0.03	0.95 ± 0.07	0.59 ± 0.04	1.02 ± 0.04	Yes
Method of Gengler et al.	0.44 ± 0.03	0.92 ± 0.07	0.58 ± 0.04	1.02 ± 0.04	Yes
Iterative peeling	0.45 ± 0.03	0.88 ± 0.05	0.60 ± 0.04	0.98 ± 0.04	Yes
All genotyped^a^	0.57 ± 0.03	0.99 ± 0.04	1	1	

Accuracy, intercept and slope of the comparison of estimated and true breeding values of 2800 females in generation 9 with phenotype but not genotype for the model including gene content (GCMTBLUP) or not (Simple BLUP), for the method of Gengler et al. [13], and for the iterative peeling method of gene prediction

^a^All individuals are genotyped

### Scenario with a major gene for a highly heritable trait

Estimates of the substitution effect (true value = 0.71) by GCMTBLUP were equal to 0.71 ± 0.04, versus 0.72 ± 0.05 obtained with the method of Gengler et al. [13] and 0.71 ± 0.05 with peeling, regardless of whether selection candidates were genotyped or not. All three methods were equally good for the prediction of the overall breeding value of the candidates (Table 4) but GCMTBLUP was slightly superior in predicting gene content. For females, BLUP was already highly accurate since females had own phenotype records (Table 5). However, GCMTBLUP was the most accurate method for predicting gene content, because it used information from the observed phenotypes at the quantitative trait.

**Table 4:** High heritability scenario for candidates to selection

Method	Quantitative trait		Gene content		Candidates genotyped
	Accuracy	Slope	Accuracy	Slope	
Simple BLUP	0.54 ± 0.02	0.99 ± 0.04	–	–	
GCMTBLUP	0.58 ± 0.02	0.99 ± 0.05	0.61 ± 0.02	1.02 ± 0.02	No
Method of Gengler et al.	0.57 ± 0.02	0.96 ± 0.06	0.57 ± 0.02	1.02 ± 0.02	No
Iterative peeling	0.57 ± 0.02	0.96 ± 0.06	0.57 ± 0.02	1.00 ± 0.02	No
GCMTBLUP	0.78 ± 0.01	1.00 ± 0.05	1	1	Yes
Method of Gengler et al.	0.77 ± 0.01	0.94 ± 0.05	1	1	Yes
Iterative peeling	0.77 ± 0.01	0.94 ± 0.05	1	1	Yes
All genotyped^a^	0.79 ± 0.01	1.00 ± 0.04	1	1	

Accuracy, intercept and slope of the comparison of estimated and true breeding values of 1315 candidates to selection for the model including gene content (GCMTBLUP) or not (Simple BLUP), for the method of Gengler et al. [13], and for the iterative peeling method of gene prediction

^a^All individuals are genotyped

**Table 5:** High heritability scenario for females with phenotype

Method	Quantitative trait		Gene content		Candidates genotyped
	Accuracy	Slope	Accuracy	Slope	
Simple BLUP	0.78 ± 0.01	1.01 ± 0.03	–	–	
GCMTBLUP	0.78 ± 0.01	1.01 ± 0.03	0.68 ± 0.02	1.02 ± 0.03	No
Method of Gengler et al.	0.78 ± 0.01	1.04 ± 0.03	0.56 ± 0.04	1.02 ± 0.03	No
Iterative peeling	0.78 ± 0.01	1.04 ± 0.03	0.57 ± 0.04	1.00 ± 0.03	No
GCMTBLUP	0.78 ± 0.01	1.01 ± 0.03	0.71 ± 0.02	1.02 ± 0.02	Yes
Method of Gengler et al.	0.78 ± 0.01	1.04 ± 0.02	0.61 ± 0.03	1.02 ± 0.02	Yes
Iterative peeling	0.78 ± 0.01	1.04 ± 0.02	0.62 ± 0.03	1.00 ± 0.02	Yes
All genotyped^a^	0.86 ± 0.01	1.00 ± 0.02	1	1	

Accuracy, intercept and slope of the comparison of estimated and true breeding values of 2800 females in generation 9 with phenotype but not genotype for the model including gene content (GCMTBLUP) or not (Simple BLUP), for the method of Gengler et al. [13], and for the iterative peeling method of gene prediction

^a^All individuals are genotyped

## Discussion

Joint analysis of genotype and phenotype data is an old problem in genetics (i.e. [10, 36–40] and many others). In general, there are three sources of information: (a) pedigree; (b) genotypes at genes or markers associated with the trait; and (c) phenotypes at the trait of interest. To infer genotypes or (in linkage studies) local genomic patterns of identity by descent across individuals, all three sources can be used in principle, i.e., the phenotype at the trait of interest can serve to infer the genotype at the gene or marker. In many cases, this is not done due to its complexity; obtaining the joint distribution of the markers and a complex trait is a notoriously difficult task that fails for complex pedigrees, even by using Monte Carlo methods [41]. As a result, a two-step procedure is often used where genotypes are used or deduced first and the phenotypes are used later [8, 9, 13, 38, 39, 42]. Also, phenotypes at the trait of interest are projected on the genotyped individuals [43, 44]. All these cases result in incomplete use of information, which is of two kinds. First, the genotype of an individual that is not genotyped is deduced without using the phenotype at the quantitative trait [13, 42]. Second, the phenotype of an individual that is not genotyped is assigned to a genotyped individual [43, 44], and this assumes that the ungenotyped individual had on average the same genotype as the genotyped individual. In the context of multiple marker genomic selection, these problems are solved using single-step genomic BLUP (GBLUP) [17, 45]. In single-step GBLUP, it is in principle possible to weight markers individually [46, 47] and, thus, it can explicitly include major genes but this has not yet been attempted with real data.

However, a simpler method that considers major genes, as proposed here, is desirable for two reasons. First, there are species for which there is no regular multiple marker genotyping of individuals but major genes exist and have been identified. This is the case for dairy goats (casein genes), sheep (several major genes for prolificacy), and pigs (halothane gene). In these species, not all animals are genotyped. Also, a single-step model including GCMTBLUP (i.e., the polygenic component is evaluated using single-step, whereas the major gene is evaluated as a correlated trait) gives a simple alternative to weighted or Bayesian regression single-step [46, 47], which might be of interest when there are only a few major genes, for instance in the case of *DGAT1* [2] for fat content in dairy cattle. The method that we developed here fulfills these conditions. First, it uses all the information and is optimal among linear estimators (in Henderson’s sense); second, it is straightforward to implement using available multi-trait BLUP software.

The GCMTBLUP method can be seen as a simple extension of the methods of Gengler et al. [13] and Forneris et al. [14], who estimated heritabilities of gene contents; here we propose to fit gene content as a correlated trait instead of a single trait. Christensen and Lund [17] used the method of Gengler et al. [13] as a starting point to develop single-step GBLUP, and they found that its problem was that missing genotypes (${\mathbf{z}}_{m}$) were fixed at their estimated values, ($\widehat{{\mathbf{z}}}_{m}$), which Christensen and Lund corrected by considering $Var(\widehat{{\mathbf{z}}}_{m} )$ as a function of relationships and heterozygosity $2pq$ at the markers. This shows how close GCMTBLUP is to single-step GBLUP. One difference is that, in GCMTBLUP, allele frequencies in the base population are explicitly included in the model and estimated, which avoids problems of compatibility that are difficult to solve for multiple markers [48, 49].

One advantage of GCMTBLUP over the method of Gengler et al. [13] or peeling [35] is that it considers trait phenotypes to infer the genotype and, thus, is in principle more accurate. Also, the estimated gene effect is not (or less) biased by selection with GCMTBLUP, because selection on the trait is accounted for. Kennedy et al. [4] reported upward bias in the estimate of the gene effect if genotyped animals were considered as “unrelated”. Peeling and gene content prediction do not use the phenotype and thus the selection process is ignored, which leads to bias [8, 9].

Regarding our simulations, it can be argued that scenarios with $p = 0.5$ are the least favorable to peeling, as the number of known transmitted alleles is minimal (i.e., 50 % of the animals in the population are heterozygous). Simulations with $p = 0.25$ for the favorable allele, however, led to very similar results, i.e. similar accuracies across methods but bias when using the method of Gengler et al. [13] or peeling to deduce genotypes.

Another advantage of GCMTBLUP that can be observed in Tables 2, 3, 4 and 5 is that prediction with GCMTBLUP is less biased, because it does not assume that the “imputed” genotype is exact. For instance, assume a sheep whose parents have genotypes AB and AB for a prolificacy gene (say BB increases prolificacy). Prediction of gene content using any method will provide estimates of the progeny genotype (either a point estimate of AB or probabilities 0.25, 0.5 and 0.25 for each class); these estimates will be constant during the life of the animal and for all subsequent BLUP analyses. However, if the sheep has repeatedly large litter sizes, the genotype is clearly BB, and GCMTBLUP can “see” this information to obtain a more accurate estimate of the genotype. Estimation of genotypes is useful not only for genetic evaluation but also for mating purposes. Meuwissen and Goddard [6] suggested an approximate hybrid method in which (approximate) iterative peeling was weighted by the likelihood given the quantitative trait, but their method is complex to implement and, to the best of our knowledge, has not been tested for genetic evaluation.

An additional advantage of GCMTBLUP over iterative peeling is that GCMTBLUP can handle very large datasets (provided the effect of the gene is known), whereas iterative peeling is not exact and slow. More refined procedures can be devised that would couple peeling with BLUP style genetic evaluation. For instance, GCMTBLUP ignores the fact that an AA sire can only produce A gametes.

We also give an extension to multiallelic genes, whereas in previous approximations [13, 31] the covariance generated by a finite number of alleles was ignored. Multi-allelism is well considered by (iterative) peeling [35, 42].

Extension to multiple genes (and multiple quantitative traits) is in principle straightforward, although the size of the equations will grow up quadratically and estimation of all required parameters will become more complex and less accurate. In principle, major genes should have non-zero genetic correlations only if they are physically linked and in linkage disequilibrium in the base population.

Although quite straightforward, we show, for the first time, the derivation of the covariance of gene content with a trait. However, it was not until recently that major genes were included in regular evaluations [9, 12]. Also, livestock pedigrees are extraordinarily complex, which precludes the use of methods such as exact peeling. Lack of availability of good software for peeling also complicates research into these methods, whereas efficient public software and, particularly, source codes exist for BLUP.

Deviations from linearity are common for the effects of major genes [3]. If the gene has a dominant action, then the substitution effect is $\alpha = a + d(q - p)$. Under selection or drift, this substitution effect changes as allele frequencies change. Thus, in practice, if there are large deviations due to dominance, the GCMTBLUP method proposed here could be used by including only data from a few generations back, so there is no major change in frequencies.

A further complication arises if one of the homozygotes (say AA) is sterile; then there is no equilibrium until allele A is lost, unless it is under selection. However, Hardy–Weinberg proportions approximately hold if $q$ is small; from one generation to another, $\Delta q \approx - q^{2}$ for small $q$ [15]. Thus, the expression for the substitution effect $\alpha = a + d(q - p)$ is approximately correct over generations, and GCMTBLUP is a good approximation. However, breeding values and substitution effects are really meaningful concepts if mating is at random. In the particular case of major genes with sterile homozygotes, other methods including dominance variation [50] or finite locus models [51] could be used.

## Conclusions

GCMTBLUP provides a single, efficient and streamlined method for genetic evaluation including polygenic effects and biallelic or multiallelic major genes when not all individuals in the population are genotyped. It considers all sources of information, it is computationally efficient, and produces unbiased estimates of gene effects and unbiased BLUP predictions. Standard software can be used.

## Appendix

### Equivalence between GCMTBLUP and fitting the genotype as a covariate when all animals are genotyped

Here we prove that when all individuals with phenotype have genotype, using a multiple trait model for $y$ and $z$, as we suggest, or fitting major genes as a fixed covariate [4] gives the same result for total genetic merit $u_{y}$. Consider a simple case, animal model with one record per individual and all individuals genotyped. The proof however extends to any setting where all individuals with phenotype have genotype. Consider centered $y$ and $z$ to make the algebra simpler. Assume that $2pq$ and $\alpha$ are known.

We have:

$${\mathbf{G}}_{0} = Var\left( {\begin{array}{*{20}c} {{\mathbf{u}}_{y} } \\ {{\mathbf{u}}_{z} } \\ \end{array} } \right) = \left( {\begin{array}{*{20}c} {\sigma_{\varepsilon }^{2} + 2pq\alpha^{2} } & {2pq\alpha } \\ {2pq\alpha } & {2pq} \\ \end{array} } \right)$$

With the inverse:

$${\mathbf{G}}_{0}^{ - 1} = \left( {\begin{array}{*{20}c} {\sigma^{11} } & {\sigma^{12} } \\ {\sigma^{21} } & {\sigma^{22} } \\ \end{array} } \right) = \frac{1}{{2pq\sigma_{\varepsilon }^{2} }}\left( {\begin{array}{*{20}c} {2pq} & { - 2pq\alpha } \\ { - 2pq\alpha } & {\sigma_{\varepsilon }^{2} + 2pq\alpha^{2} } \\ \end{array} } \right) = \left( {\begin{array}{*{20}c} {1/\sigma_{\varepsilon }^{2} } & { - \alpha /\sigma_{\varepsilon }^{2} } \\ { - \alpha /\sigma_{\varepsilon }^{2} } & {\frac{{\sigma_{\varepsilon }^{2} + 2pq\alpha^{2} }}{{2pq\sigma_{\varepsilon }^{2} }}} \\ \end{array} } \right)$$

If we know the effect of the gene, $\alpha$, then the prediction for the quantitative trait is simply:

$$\widehat{{\mathbf{u}}}_{y} = \widehat{\boldsymbol{{\varepsilon}}} + {\mathbf{z}}\alpha = ({\mathbf{I}}\sigma_{ey}^{ - 2} + {\mathbf{A}}^{ - 1} \sigma_{\varepsilon }^{ - 2} )^{ - 1} ({\mathbf{y}} - {\mathbf{z}}\alpha )\sigma_{ey}^{ - 2} + {\mathbf{z}}\alpha ,$$

$$({\mathbf{I}}\sigma_{ey}^{ - 2} + {\mathbf{A}}^{ - 1} \sigma_{\varepsilon }^{ - 2} )^{ - 1} ({\mathbf{y}} - {\mathbf{z}}\alpha )\sigma_{ey}^{ - 2} + {\mathbf{z}}\alpha = ({\mathbf{I}}\sigma_{ey}^{ - 2} + {\mathbf{A}}^{ - 1} \sigma_{\varepsilon }^{ - 2} )^{ - 1} [({\mathbf{y}} - {\mathbf{z}}\alpha )\sigma_{ey}^{ - 2} + ({\mathbf{I}}\sigma_{ey}^{ - 2} + {\mathbf{A}}^{ - 1} \sigma_{\varepsilon }^{ - 2} ){\mathbf{z}}\alpha ].$$

We expand the part inside the square brackets:

$$\left[ {\left( {{\mathbf{y}} - {\mathbf{z}}\alpha } \right)\sigma_{ey}^{ - 2} + \left( {{\mathbf{I}}\sigma_{ey}^{ - 2} + {\mathbf{A}}^{ - 1} \sigma_{\varepsilon }^{ - 2} } \right){\mathbf{z}}\alpha } \right] = {\mathbf{y}}\sigma_{ey}^{ - 2} - {\mathbf{z}}\alpha \sigma_{ey}^{ - 2} + {\mathbf{z}}\alpha \sigma_{ey}^{ - 2} + {\mathbf{A}}^{ - 1} \sigma_{\varepsilon }^{ - 2} {\mathbf{z}}\alpha = {\mathbf{y}}\sigma_{ey}^{ - 2} + {\mathbf{A}}^{ - 1} \sigma_{\varepsilon }^{ - 2} {\mathbf{z}}\alpha .$$

So, we can write that the estimator of the total genetic value is:

1 $$({\mathbf{I}}\sigma_{ey}^{ - 2} + {\mathbf{A}}^{ - 1} \sigma_{\varepsilon }^{ - 2} )\widehat{{\mathbf{u}}}_{y} = {\mathbf{y}}\sigma_{ey}^{ - 2} + {\mathbf{A}}^{ - 1} \sigma_{\varepsilon }^{ - 2} {\mathbf{z}}\alpha .$$

Construct the two trait mixed model equations (note that $\sigma_{ey,z} = 0$):

$$\left( {\begin{array}{*{20}c} {{\mathbf{I}}\sigma_{ey}^{ - 2} + {\mathbf{A}}^{ - 1} \sigma^{11} } & {{\mathbf{A}}^{ - 1} \sigma^{12} } \\ {{\mathbf{A}}^{ - 1} \sigma^{21} } & {{\mathbf{A}}^{ - 1} \sigma^{22} + {\mathbf{I}}\sigma_{ez}^{ - 2} } \\ \end{array} } \right)\left( {\begin{array}{*{20}c} {\widehat{{{\mathbf{u}}_{y} }}} \\ {\widehat{{{\mathbf{u}}_{z} }}} \\ \end{array} } \right) = \left( {\begin{array}{*{20}c} {{\mathbf{y}}\sigma_{ey}^{ - 2} } \\ {{\mathbf{z}}\sigma_{ez}^{ - 2} } \\ \end{array} } \right).$$

Absorb the equations for $\widehat{{\mathbf{u}}}_{z}$:

$$\left( {{\mathbf{I}}\sigma_{ey}^{ - 2} + {\mathbf{A}}^{ - 1} \sigma^{11} - {\mathbf{A}}^{ - 1} \sigma^{21} \left( {{\mathbf{A}}^{ - 1} \sigma^{22} + {\mathbf{I}}\sigma_{ez}^{ - 2} } \right)^{ - 1} {\mathbf{A}}^{ - 1} \sigma^{12} } \right)\widehat{{{\mathbf{u}}_{y} }}
= {\mathbf{y}}\sigma_{ey}^{ - 2} - {\mathbf{A}}^{ - 1} \sigma^{21} \left( {{\mathbf{A}}^{ - 1} \sigma^{22} + {\mathbf{I}}\sigma_{ez}^{ - 2} } \right)^{ - 1} {\mathbf{z}}\sigma_{ez}^{ - 2} .$$

When $\sigma_{ez}^{2} \to 0$ (the true value of residual variance for gene content) then:

$$({\mathbf{A}}^{ - 1} \sigma^{22} + {\mathbf{I}}\sigma_{ez}^{ - 2} )^{ - 1} \to 0$$

and

$${\mathbf{A}}^{ - 1} \sigma^{21} ({\mathbf{A}}^{ - 1} \sigma^{22} + {\mathbf{I}}\sigma_{ez}^{ - 2} )^{ - 1} {\mathbf{z}}\sigma_{ez}^{ - 2} = {\mathbf{A}}^{ - 1} \sigma^{21} ({\mathbf{A}}^{ - 1} \sigma^{22} \sigma_{ez}^{ - 2} + {\mathbf{I}})^{ - 1} {\mathbf{z}} \to {\mathbf{A}}^{ - 1} \sigma^{21} {\mathbf{z}},$$

so

$$({\mathbf{I}}\sigma_{ey}^{ - 2} + {\mathbf{A}}^{ - 1} \sigma^{11} )\widehat{{{\mathbf{u}}_{y} }} = {\mathbf{y}}\sigma_{ey}^{ - 2} - {\mathbf{A}}^{ - 1} \sigma^{21} {\mathbf{z}}.$$

However $\sigma^{11} = 1/\sigma_{\varepsilon }^{2}$ and $\sigma^{21} = - \alpha/\sigma_{\varepsilon }^{2}$ and therefore this is equal to expression (1):

$$({\mathbf{I}}\sigma_{ey}^{ - 2} + {\mathbf{A}}^{ - 1} \sigma_{\varepsilon }^{ - 2} )\widehat{{{\mathbf{u}}_{y} }} = {\mathbf{y}}\sigma_{ey}^{ - 2} + {\mathbf{A}}^{ - 1} \sigma_{\varepsilon }^{ - 2} {\mathbf{z}}\alpha$$

which completes the proof.
